# Supplementary material for: The Performance of Miscanthus Seeds During Long-Term Storage
Source: Plants (Basel). 2025 Dec 8;14(24):3738. doi: 10.3390/plants14243738 (PMC12737046; doi:10.3390/plants14243738)
Supplement: Supplementary file 1 [file plants-14-03738-s001.zip › plants-3987817-supplementary.pdf]

Supplementary Table S1. Germination percentage (± s.e.m.) and vigor index (± s.e.m.) of seeds before storage and after storage for different durations at different storage conditions.

Genotype	Storage condition	Storage duration (years)	Germination percentage (%)	Vigor index
B0129	RT	0	86.00±1.15a	274.91±6.69a
		4	0b	0b
		5	0b	0b
	RTD	0	86.00±1.15a	274.91±6.69a
		4	62.22±5.88b	76.54±8.35b
		5	40.00±3.85c	45.25±3.56c
	RTV	0	86.00±1.15a	274.91±6.69a
		4	0b	0b
		5	0b	0b
	LT	0	86.00±1.15a	274.91±6.69a
		4	73.33±3.85a	133.13±6.40b
		5	67.78±6.19a	109.75±13.76b
	LTD	0	86.00±1.15a	274.91±6.69a
		4	80.00±6.94a	151.13±13.70b
		5	75.56±7.78a	118.94±10.74b
	LTV	0	86.00±1.15a	274.91±6.69a
		4	67.78±7.78a	116.52±10.45b
		5	68.89±4.01a	129.11±10.05b
C0421	RT	0	88.67±3.53a	196.19±6.14a
		4	0b	0b
		5	0b	0b
	RTD	0	88.67±3.53a	196.19±6.14a
		4	22.22±2.22b	14.69±1.78b
		5	12.22±4.01b	4.10±1.20b
	RTV	0	88.67±3.53a	196.19±6.14a
		4	0b	0b
		5	0b	0b
	LT	0	88.67±3.53a	196.19±6.14a
		4	45.56±4.84b	76.34±6.66b
		5	34.44±4.01b	67.84±8.96b
	LTD	0	88.67±3.53a	196.19±6.14a
		4	56.67±3.85b	92.94±6.59b
		5	40.00±3.33b	56.21±7.07c
	LTV	0	88.67±3.53a	196.19±6.14a
		4	45.56±5.88b	90.73±9.09b
		5	35.56±6.19b	52.40±10.55b
C0615	RT	0	78.00±3.06a	138.76±4.59a
		4	0b	0b

		5	0b	0b
	RTD	0	78.00±3.06a	138.76±4.59a
		4	16.67±1.92b	8.28±0.85b
		5	0c	0b
	RTV	0	78.00±3.06a	138.76±4.59a
		4	0b	0b
		5	0b	0b
	LT	0	78.00±3.06a	138.76±4.59a
		4	48.89±11.76a	98.69±19.98a
		5	12.22±1.11b	15.26±2.20b
	LTD	0	78.00±3.06a	138.76±4.59a
		4	32.22±2.22b	37.50±2.22b
		5	14.44±2.22c	13.58±3.21c
	LTV	0	78.00±3.06a	138.76±4.59a
		4	32.22±1.11b	62.65±4.20b
		5	13.33±1.92c	14.94±1.85c
D0115	RT	0	80.00±5.29a	160.29±5.69a
		4	0b	0b
		5	0b	0b
	RTD	0	80.00±5.29a	160.29±5.69a
		4	15.56±2.94b	21.63±3.58b
		5	8.89±4.84b	5.54±2.98b
	RTV	0	80.00±5.29a	160.29±5.69a
		4	0b	0b
		5	0b	0b
	LT	0	80.00±5.29a	160.29±5.69a
		4	40.00±3.33b	87.56±6.03b
		5	34.44±2.94b	68.65±4.35b
	LTD	0	80.00±5.29a	160.29±5.69a
		4	33.33±1.93b	75.92±3.34b
		5	22.22±2.22b	23.28±3.33c
	LTV	0	80.00±5.29a	160.29±5.69a
		4	26.67±1.92b	60.17±2.88b
		5	32.22±4.84b	64.14±9.17b
Y0101	RT	0	92.00±1.15a	331.27±2.45a
		4	0b	0b
		5	0b	0b
	RTD	0	92.00±1.15a	331.27±2.45a
		4	11.11±4.84b	2.45±1.10b
		5	6.67±3.85b	1.03±0.60b
	RTV	0	92.00±1.15a	331.27±2.45a
		4	0b	0b
		5	0b	0b
	LT	0	92.00±1.15a	331.27±2.45a

		4	57.78±1.11b	133.82±8.46b
		5	32.22±5.88c	47.43±9.66c
	LTD	0	92.00±1.15a	331.27±2.45a
		4	48.89±5.88b	101.04±18.0b
		5	25.56±7.78b	22.31±6.60c
	LTV	0	92.00±1.15a	331.27±2.45a
		4	51.11±9.09b	116.38±17.80b
		5	42.22±2.22b	44.01±1.73c
Z0101	RT	0	70.67±12.35a	125.25±22.77a
		4	0b	0b
		5	0b	0b
	RTD	0	70.67±12.35a	125.25±22.77a
		4	31.11±5.88b	27.59±4.55b
		5	23.33±1.92b	16.86±1.09b
	RTV	0	70.67±12.35a	125.25±22.77a
		4	0b	0b
		5	0b	0b
	LT	0	70.67±12.35a	125.25±22.77b
		4	91.11±1.11a	262.82±6.69a
		5	65.56±4.01a	173.64±9.98b
	LTD	0	70.67±12.35a	125.25±22.77b
		4	75.56±4.84a	211.28±14.77a
		5	76.67±6.94a	163.74±15.62ab
	LTV	0	70.67±12.35a	125.25±22.77a
		4	50.00±3.85a	127.84±11.22a
		5	46.67±3.33a	105.31±6.72a

Different lowercase letters denote statistical difference between storage durations of each storage condition at the $P<0.05$ level according to Duncan test. RT, seeds were stored under room temperature condition without treatment; RTD, seeds were stored under room temperature with desiccant condition; RTV, seeds were stored under room temperature with vacuum condition; LT, seeds were stored under low temperature condition with no treatment; LTD, seeds were stored under low temperature with desiccant condition; LTV, seeds were stored under low temperature with vacuum condition.

Supplementary Table S2. Emergence percentage (± s.e.m.), survival (± s.e.m.), shoot length (± s.e.m.), and dry biomass of seeds stored under different storage conditions after field sowing in the fifth year of storage.

Genotype	Storage condition	Emergence percentage (%)	Survival (%)	Shoot length (cm)	Dry biomass (g)
B0129	RTD	4.44±2.94a	0a	0b	0a
	LT	14.44±4.84a	4.44±4.44a	43.13±7.09a	1.81±1.02a
	LTD	17.78±2.94a	5.56±5.56a	46.85±5.09a	1.94±1.08a
	LTV	12.22±4.01a	10.00±5.09a	35.80±5.99a	0.83±0.30a
C0421	RTD	1.11±1.11a	0	0	0
	LT	4.44±2.22a	0	0	0
	LTD	5.56±2.22a	0	0	0
	LTV	5.56±2.94a	0	0	0
C0615	RTD	1.11±1.11a	0	0	0
	LT	0a	0	0	0
	LTD	1.11±1.11a	0	0	0
	LTV	0a	0	0	0
D0115	RTD	0a	0	0	0
	LT	2.22±1.11a	0	0	0
	LTD	4.44±1.11a	0	0	0
	LTV	1.11±1.11a	0	0	0
Y0101	RTD	0a	0a	0c	0b
	LT	5.56±2.94a	3.33±3.33a	60.45±3.81a	2.45±0.29a
	LTD	10.00±6.67a	8.89±7.29a	41.92±1.43b	0.81±0.09ab
	LTV	7.78±2.94a	4.44±4.44a	56.09±6.29a	2.14±0.86a
Z0101	RTD	1.11±1.11a	0a	0b	0a
	LT	14.44±8.01a	8.89±5.88a	37.44±4.00a	1.27±0.51a
	LTD	10.00±3.85a	8.89±4.84a	36.76±5.64a	1.03±0.31a
	LTV	7.78±4.44a	5.56±2.22a	48.40±6.35a	1.89±0.93a

None of the germinated seeds were stored under RT and RTV; hence, they were omitted. RT, seeds were stored under room temperature condition without treatment; RTD, seeds were stored under room temperature condition with desiccant; RTV, seeds were stored under room temperature with vacuum condition; LT, seeds were stored under low temperature condition without treatment; LTD, seeds were stored under low temperature condition with desiccant; LTV, seeds were stored under low temperature with vacuum condition.

Supplementary Table S3. Geographic locations for six *Miscanthus* individuals.

Genotype	Species	Source	Latitude (° N)	Longitude (°E)	Altitude (m)
B0129	*M. lutarioriparius*	Huanggang	29.79	115.97	25
C0421	*M. sinensis*	Fushun	41.95	125.10	516
C0615	*M. sacchariflorus*	Zibo	36.28	118.02	509
D0115	*M. sinensis*	Wenzhou	27.83	121.17	12
Y0101	*M. lutarioriparius*	Yuanjiang	28.84	112.35	36
Z0101	Hybrid	*M. lutarioriparius* × *M. sinensis*	-	-	-

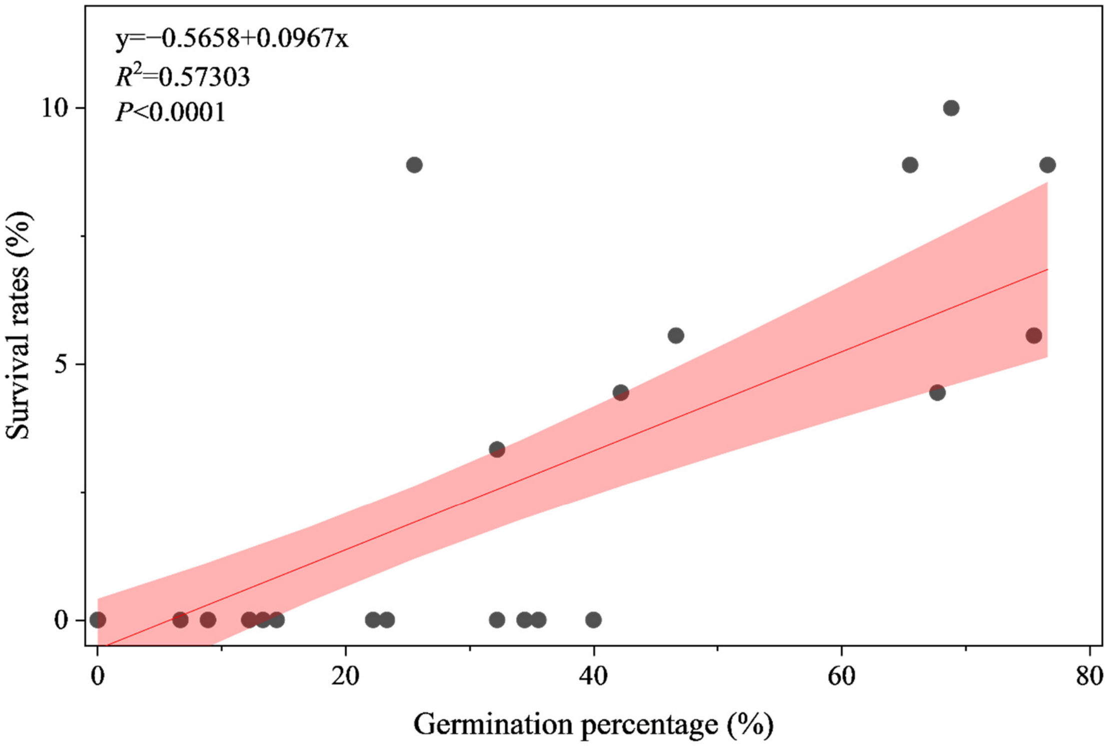

Supplementary Figure S1. Correlation between germination percentage and field survival after 5 years of storage. The red shadow of linear regressions denotes the 95% confidence interval.

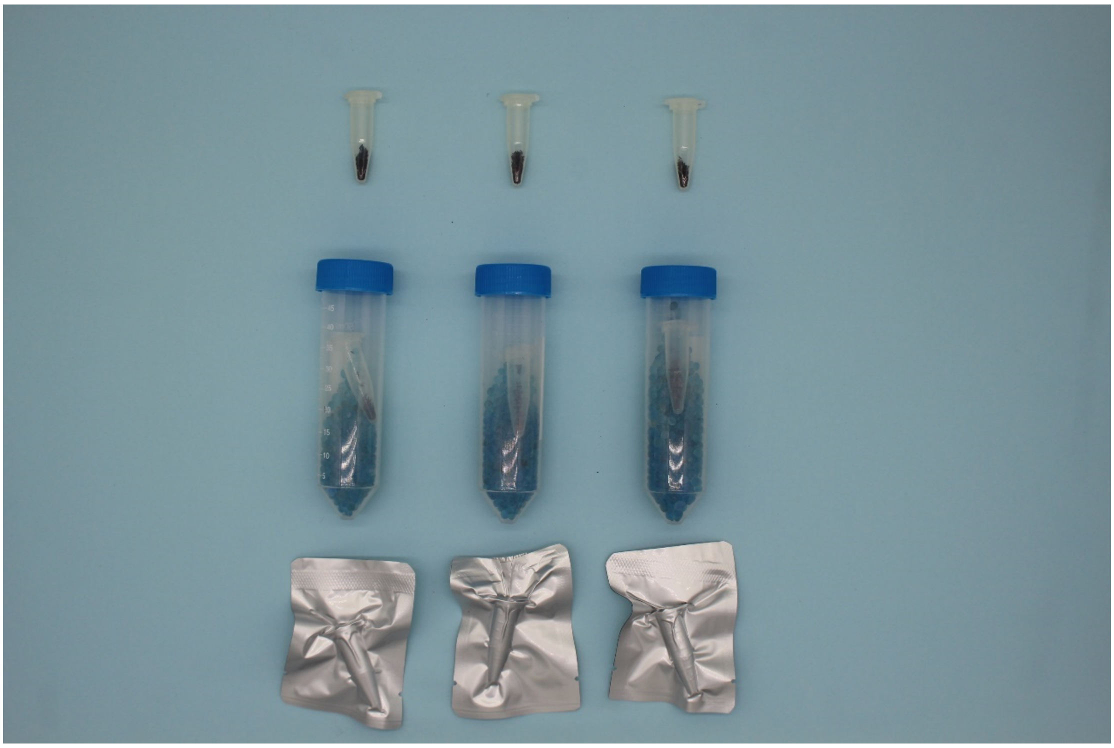

Supplementary Figure S2. From top to bottom: untreated, desiccant, and vacuum treatments.

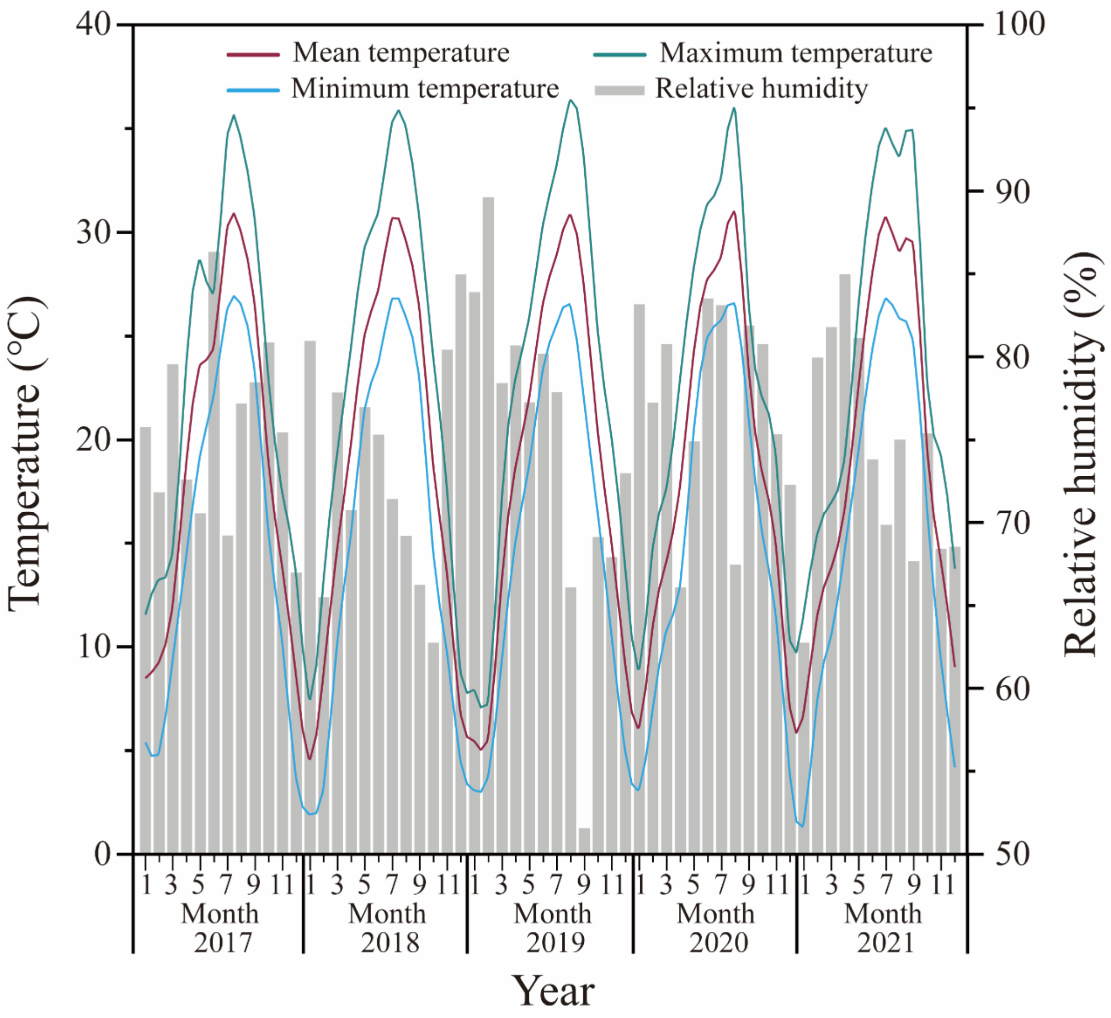

Supplementary Figure S3. Minimum, mean, and maximum air temperature (lines) and relative humidity (columns) during the 5-year storage period at the Changsha experiment site.
